# Acute Foot Drop Due to Common Peroneal Neuropathy After Primary Total Knee Arthroplasty: A Case Report With Complete Recovery

**DOI:** 10.1155/cro/9751384

**Published:** 2026-10-06

**Authors:** Sayyaf Abdullah Najmi, Yahya Mohamed Faqihi, Ibrahim M. Mahmoud, Bandar Ali Albariqi, Mohaned Mohamed Sanousi

**Affiliations:** ^1^ Orthopedic Surgery Department Armed Forces Hospital Jazan Jazan Saudi Arabia

**Keywords:** case report, common peroneal nerve palsy, conservative management, foot drop, functional recovery, nerve injury, rehabilitation, total knee arthroplasty

## Abstract

**Background:**

Common peroneal nerve palsy (CPNP) is an uncommon but potentially disabling complication of primary total knee arthroplasty (TKA). It typically presents with weakness of ankle and toe dorsiflexion, foot drop, a steppage gait and reduced or absent sensation over the dorsum of the foot. Delayed recognition or inadequate management may lead to atrophy of the anterior and lateral compartment muscles and persistent disability.

**Case presentation:**

We report a 72‐year‐old woman who developed an acute, complete CPNP with foot drop and dorsal foot sensory loss on the first day after primary right TKA. Management was nonoperative and centred on early limb repositioning, ankle‐foot orthosis (AFO) support and a structured physiotherapy programme that included neuromuscular electrical (faradic) stimulation. Full neurological recovery was achieved 115 days after surgery, with normal muscle power and no residual sensory deficit.

**Conclusion:**

Complete CPNP after TKA carries a less favourable prognosis than incomplete palsy, yet this patient regained full function within approximately 4 months of conservative care underscoring that, in the absence of a compressive lesion or progressive deficit, early nonoperative management with orthotic support and physiotherapy can achieve excellent recovery and that surgical exploration can reasonably be reserved for deterioration or failure to improve.

## 1. Introduction

Common peroneal nerve palsy (CPNP) is a rare but potentially debilitating complication of total knee arthroplasty (TKA) [1, 2]. The reported incidence ranges from 0.3% to 4% following primary TKA [1, 3]. Recognised risk factors include intraoperative correction of a valgus or flexion deformity, prolonged tourniquet time, epidural anaesthesia, elevated body mass index (BMI), local nerve compression (for example by a haematoma or pseudotumour) and pre‐existing spinal pathology [3–6].

Most cases are identified within 48 h of surgery, as the injury usually occurs during or shortly after the procedure. Reported rates of full recovery vary widely, from 9.1% to 68%, and depend strongly on whether the initial palsy is complete or incomplete [5, 7]. We present a case of acute complete CPNP after primary TKA that achieved full recovery with conservative management, and we discuss the clinical reasoning, electrodiagnostic interpretation and the factors that may have contributed to this favourable outcome. This case is reported in accordance with the CARE guidelines, and the completed CARE checklist is provided as File S1 (see Supporting Information).

## 2. Case Presentation

A 72‐year‐old woman presented with several years of right knee pain, progressive deformity and functional limitation due to severe osteoarthritis. Weight‐bearing radiographs demonstrated grade IV (Kellgren–Lawrence) osteoarthritis of the medial compartment of the right knee (Figure 1). Preoperative alignment showed a mild varus deformity. On clinical examination, the right knee had a fixed flexion deformity of approximately 15°. Her medical history included hypertension, chronic low back pain with lumbar radiculopathy and previously diagnosed carpal tunnel syndrome. These were documented as separate, focal conditions; no generalised peripheral neuropathy had been diagnosed, although pre‐existing focal neural compromise has been proposed as a possible susceptibility factor. Conservative treatment with analgesia and physiotherapy had failed to relieve her symptoms, and surgery was therefore planned.

**Figure 1:**
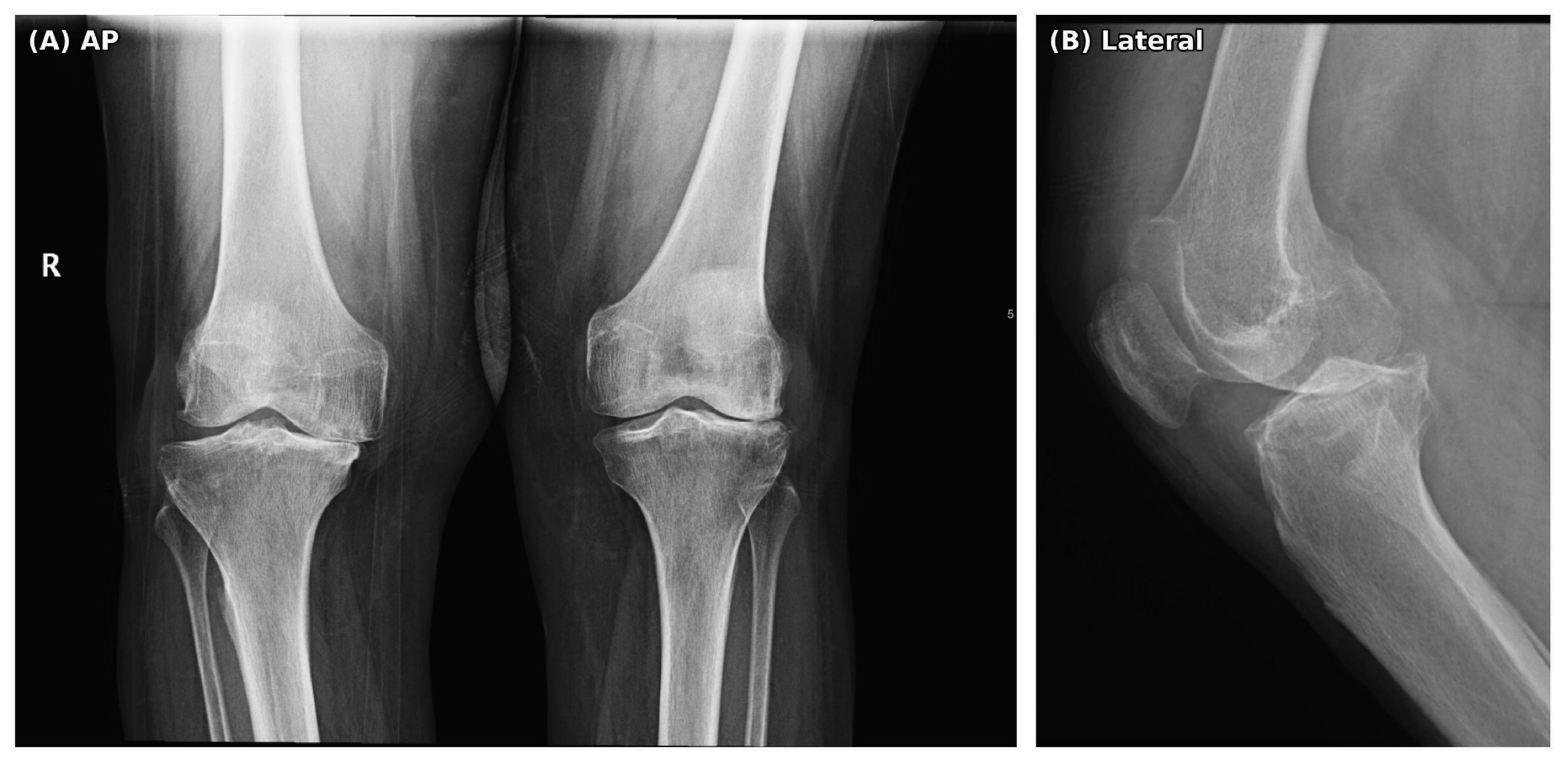
Preoperative radiographs of the right knee. (A) Anteroposterior (AP) view; (B) lateral view. The AP view shows grade IV medial‐compartment osteoarthritis with mild varus alignment (right side marked “R”).

On preoperative assessment her height was 148 cm, weight 64 kg and BMI 29.2 kg/m^2^. She was taking amlodipine; aspirin was withheld 5 days before surgery. On the day of surgery (Day 0**)**, she underwent right TKA following counselling and informed consent. Anaesthesia was spinal. A cemented, posterior‐stabilised prosthesis was implanted (Figure 2). A pneumatic thigh tourniquet was applied to the upper thigh and inflated to 350 mmHg for 120 min. The procedure was completed without intraoperative complication.

**Figure 2:**
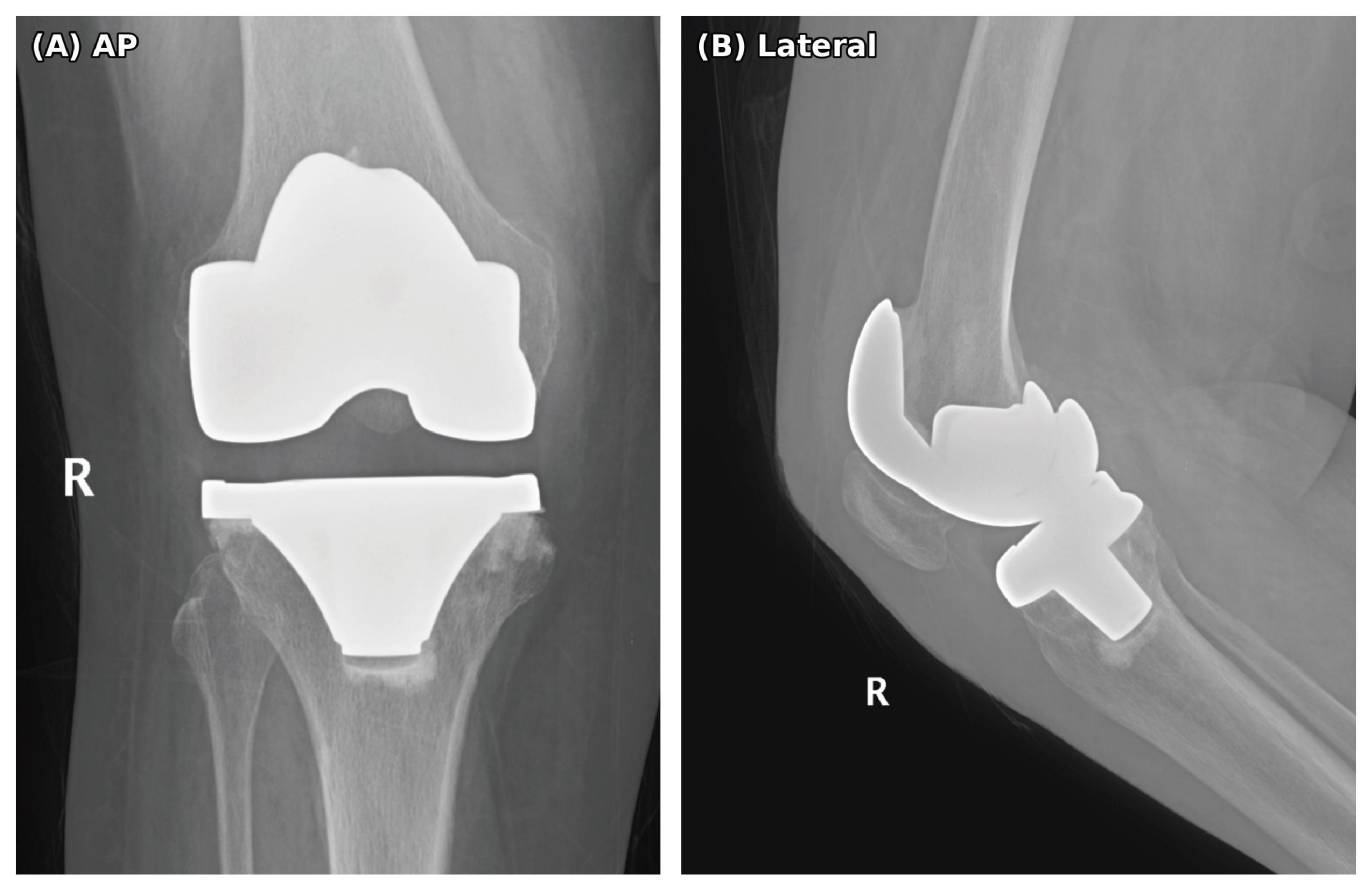
Postoperative radiographs of the right knee following primary TKA. (A) AP view; (B) lateral view, showing a well‐fixed cemented posterior‐stabilised implant (right side marked “R”).

On the first postoperative day, the patient developed acute foot drop with sensory disturbance in the superficial and deep peroneal distribution of the operated limb. The palsy was complete: manual muscle testing (MMT) showed Grade 0/5 power in ankle dorsiflexion, great‐toe extension and foot eversion, whereas plantar flexion and inversion were preserved at 5/5 (Table 1). Sensation over the dorsum of the foot was diminished and subsequently absent, involving both the superficial peroneal (dorsolateral foot) and deep peroneal (first dorsal web space) sensory territories. Distal pulses were palpable, and there was no evidence of vascular compromise, compartment syndrome or excessive postoperative swelling.

**Table 1:** Manual muscle testing (MMT, 0–5) over the clinical course.

Muscle/action (nerve)	Day 1	Day 91	Day 115
Ankle dorsiflexion—tibialis anterior (deep peroneal)	0/5	3/5	5/5
Great‐toe extension—EHL (deep peroneal)	0/5	3/5	5/5
Foot eversion—peronei (superficial peroneal)	0/5	3/5	5/5
Ankle plantar flexion (tibial)	5/5	5/5	5/5
Foot inversion—tibialis posterior (tibial)	5/5	5/5	5/5

Abbreviation: EHL, extensor hallucis longus.

Conservative management was started immediately. Compressive dressings were removed, and the limb was positioned with the hip in neutral extension (0°) and the knee flexed to approximately 30° to reduce tension on the nerve. An ankle‐foot orthosis (AFO) was fitted on postoperative Day 2 to prevent an equinus deformity and to assist ambulation. No compressive haematoma was identified clinically, and there were no features of compartment syndrome or vascular compromise. Surgical exploration was not indicated: no compressive lesion was identified, there was no progressive neurological deficit and spontaneous recovery was anticipated. Conservative management was therefore adopted as the appropriate first‐line treatment, with surgical exploration reserved for any subsequent deterioration or failure to recover.

Motor nerve conduction studies were performed on postoperative Day 42 (6 weeks), timed to allow any Wallerian degeneration to become electrophysiologically detectable and to characterise the lesion once acute changes had evolved. Only motor studies were undertaken; sensory conduction and needle electromyography were not performed. The right common peroneal motor study (recording over extensor digitorum brevis) showed no recordable response even with maximal stimulation, whereas the right tibial motor study was preserved (distal latency 3.6 ms; distal amplitude 4.0 mV; proximal [popliteal] latency 11.1 ms; conduction velocity 44 m/s).

A structured physiotherapy programme was delivered as two 60‐min sessions per week for 10 weeks. It comprised neuromuscular electrical (faradic) stimulation, ankle dorsiflexion strengthening, joint range‐of‐motion maintenance and gait training with full weight‐bearing, with the AFO maintained to aid ambulation and prevent falls. Muscle strength and activation were reviewed every 2 weeks. At postoperative Day 91 (approximately 3 months), ankle dorsiflexion had improved to Grade 3/5, indicating recovery of active movement against gravity, although weakness of great‐toe extension persisted; the patient continued to mobilise with an AFO and a walking cane. By postoperative Day 115 (approximately 4 months), she had achieved full neurological recovery, with 5/5 power across all previously affected muscle groups and restored active ankle motion (dorsiflexion +10–20° and plantar flexion 40–50° from a neutral position), confirming resolution of the foot drop. She had recovered a normal gait pattern, a functional range of knee motion from full extension (0°) to 90° of flexion and no residual sensory deficit. The clinical course from surgery to full recovery is summarised in Table 2.

**Table 2:** Structured clinical timeline (days from surgery).

Time point	Event/findings
Day 0	Primary right TKA; spinal anaesthesia; thigh tourniquet 350 mmHg for 120 min.
Day 1	Acute complete CPNP: dorsiflexion, great‐toe extension and eversion 0/5; dorsal foot sensory loss; tibial function preserved. Compressive dressings removed; limb repositioned (hip 0°, knee 30°).
Day 2	AFO fitted; structured physiotherapy commenced (faradic stimulation, ROM, gait training).
Day 42 (6 weeks)	Motor NCS: unrecordable common peroneal response; preserved tibial study. Sensory/EMG not performed.
Day 91 (~3 months)	Partial recovery: dorsiflexion 3/5; mobilising with AFO and cane.
Day 115 (~4 months)	Full recovery: 5/5 across affected groups; active ankle dorsiflexion restored (+10–20°); normal gait; no residual sensory deficit.

Abbreviations: AFO, ankle‐foot orthosis; CPNP, common peroneal nerve palsy; NCS, nerve conduction study; ROM, range of motion.

### 2.1. Patient Perspective

From the patient′s perspective, the unexpected foot drop was initially concerning, but she was reassured by the steady recovery and was satisfied with her final outcome.

## 3. Discussion

The common peroneal nerve is particularly vulnerable at the fibular neck, where its superficial course exposes it to traction, compression, or ischaemic injury during and after knee surgery [2, 8]. In the present case, several recognised contributors were present—advanced age, an elevated BMI (29.2 kg/m^2^), a prolonged tourniquet time (120 min) and a preoperative fixed flexion deformity of approximately 15°, the intraoperative correction of which is a recognised risk factor for peroneal nerve palsy because it lengthens the posterior soft tissues and increases tension on the nerve. By contrast, two other classic factors were absent: the coronal deformity was varus rather than valgus, and she received spinal rather than epidural anaesthesia [6, 9]. This combination suggests that correction of the flexion deformity, together with tourniquet‐related and patient‐related factors, was more relevant than neuraxial anaesthesia in this instance.

The diagnosis was established on clinical grounds and localised to the common peroneal nerve at the fibular neck. The acute onset immediately after surgery on the operated side, with deficits confined to the peroneal distribution (weak dorsiflexion, toe extension and eversion, with dorsal foot sensory loss) and preserved tibial function (intact plantar flexion and inversion), argued against alternative causes. Lumbar (L5) radiculopathy was considered given her back history but was thought unlikely, as tibialis posterior (L5, tibial nerve) inversion was preserved and the deficit was acute and clearly related to surgery; a lumbosacral plexopathy would be expected to involve nerve territories beyond the peroneal distribution; and anaesthesia‐related neuropathy was improbable given the focal, unilateral peroneal pattern following spinal anaesthesia. Preserved tibial conduction and palpable distal pulses further excluded a global neurovascular or compartment problem.

The electrodiagnostic findings warrant careful interpretation. An unrecordable motor response does not, by itself, establish a conduction block, since an absent compound muscle action potential may reflect either a demyelinating block or axonal loss; the two cannot be distinguished on an absent motor response alone, and sensory studies and needle EMG were not available in this case. The injury therefore could not be definitively graded. Nonetheless, the subsequent rapid and complete clinical recovery is most consistent with a predominantly neurapraxic (conduction‐block) lesion, in which an early absent response can be followed by full recovery.

The educational value of this case lies in its favourable course despite an initially complete palsy. Complete CPNP after TKA is generally associated with a poorer prognosis than incomplete palsy. In a systematic review, complete palsies recovered fully in approximately 39% of cases versus 66% for incomplete palsies [7], and reported times to full recovery commonly extend over many months [5, 10, 11]. Against this background, the achievement of full recovery within 115 days in a patient with a complete palsy is noteworthy. Factors that may have contributed include the prompt removal of external compression, early limb repositioning, consistent orthotic support and a structured physiotherapy programme incorporating neuromuscular electrical stimulation, together with close monitoring and patient education throughout.

Consistent with the literature, conservative management is appropriate for CPNP after TKA in the absence of a progressive deficit or a compressive lesion [4, 12]. Surgical exploration or decompression is generally reserved for deterioration or failure to recover over time [12]. In the present case, conservative treatment was the appropriate first‐line approach because no compressive lesion was identified and there was no progressive deficit; her outcome supports this approach. Causality should nonetheless be interpreted cautiously: recovery occurred during conservative management and cannot be attributed to it with certainty, as Sunderland Grade I–II injuries may recover as part of their natural history [13].

### 3.1. Limitations

This is a single case, which limits generalisability. Electrodiagnostic assessment was restricted to motor conduction; sensory studies and needle EMG were not performed, so the lesion could not be formally graded. Coronal alignment was estimated on standard rather than full‐length weight‐bearing radiographs.

## 4. Conclusion

CPNP is an uncommon but potentially disabling complication of TKA that should be recognised early and managed promptly. This case demonstrates that even a complete palsy can recover fully with conservative measures relief of external compression, orthotic support and a structured physiotherapy programme when no compressive lesion is present. Awareness of patient‐related and surgery‐related risk factors supports prevention and early diagnosis, and a high index of suspicion in the immediate postoperative period offers the best chance of an excellent functional outcome while avoiding unnecessary surgery.

## Author Contributions

All authors contributed to patient care, data collection, drafting and critical revision of the manuscript.

## Funding

No funding was received for this manuscript.

## Disclosure

All authors approved the final version of the manuscript.

## Ethics Statement

Ethical approval was not required for this case report in accordance with institutional policy.

## Consent

Written informed consent was obtained from the patient for publication of this case report and the accompanying images.

## Conflicts of Interest

The authors declare no conflicts of interest.

## Supporting information


**Supporting Information** Additional supporting information can be found online in the Supporting Information section. Supporting Information. File S1: CARE checklist: the completed CARE (CAse REport) reporting guideline checklist, indicating the section of the manuscript in which each item is addressed.
