# Supplementary material for: Acute Foot Drop Due to Common Peroneal Neuropathy After Primary Total Knee Arthroplasty: A Case Report With Complete Recovery
Source: Case Rep Orthop. 2026 Oct 6;2026:9751384. doi: 10.1155/cro/9751384 (PMC13643216; doi:10.1155/cro/9751384)
Supplement: Supplementary file 1 [file CRO-2026-9751384-s001.docx]

**CARE Checklist (2016)**

Manuscript ID 9751384 — Case Reports in Orthopedics

*Acute Foot Drop Due to Common Peroneal Neuropathy After Primary Total Knee Arthroplasty: A Case Report With Complete Recovery*

This checklist confirms that the manuscript reports each element recommended by the CARE (CAse REport) guidelines. The final column indicates where each item is addressed.

| **#** | **CARE checklist item** | **Reported** | **Location in manuscript** |
| --- | --- | --- | --- |
| 1 | Title — the area of focus and “case report” | Yes | Title page |
| 2 | Key words — 2–5 key words | Yes | Abstract (Keywords) |
| 3a | Abstract — background | Yes | Abstract (Background) |
| 3b | Abstract — main symptoms and clinical findings | Yes | Abstract (Case presentation) |
| 3c | Abstract — diagnoses, interventions, outcomes | Yes | Abstract (Case presentation) |
| 3d | Abstract — conclusion | Yes | Abstract (Conclusion) |
| 4 | Introduction — brief background with references | Yes | Section 1 (Introduction) |
| 5a | Patient information — de-identified demographics | Yes | Section 2, paragraph 1 |
| 5b | Patient information — main concerns and symptoms | Yes | Section 2, paragraphs 1 and 3 |
| 5c | Patient information — medical, family, psychosocial history | Yes | Section 2, paragraph 1 |
| 5d | Patient information — relevant past interventions and outcomes | Yes | Section 2, paragraph 1 |
| 6 | Clinical findings — relevant physical examination findings | Yes | Section 2, para 3; Table 1 (MMT) |
| 7 | Timeline — organised as a timeline | Yes | Table 2 (clinical timeline) |
| 8a | Diagnostic assessment — methods (examination, NCS) | Yes | Section 2; Supplementary Figure 1 |
| 8b | Diagnostic assessment — diagnostic challenges | Yes | Section 3 (electrodiagnostic interpretation) |
| 8c | Diagnostic assessment — diagnostic reasoning / differential | Yes | Section 3 (differential diagnosis) |
| 8d | Diagnostic assessment — prognostic characteristics | Yes | Section 3 (complete vs incomplete palsy) |
| 9a | Therapeutic intervention — types | Yes | Section 2 (positioning, AFO, physiotherapy) |
| 9b | Therapeutic intervention — administration (dose, duration) | Yes | Section 2 (physiotherapy programme) |
| 9c | Therapeutic intervention — changes with rationale | Yes | Section 2 (management course) |
| 10a | Follow-up and outcomes — clinician- and patient-assessed outcomes | Yes | Section 2; Table 1 |
| 10b | Follow-up and outcomes — important follow-up findings | Yes | Section 2 (3-month and final review) |
| 10c | Follow-up and outcomes — adherence and tolerability | Yes | Section 2 (physiotherapy) |
| 10d | Follow-up and outcomes — adverse events | Yes | Section 2 (none reported) |
| 11a | Discussion — strengths and limitations | Yes | Section 3 (Limitations) |
| 11b | Discussion — relevant medical literature | Yes | Section 3 |
| 11c | Discussion — rationale for conclusions | Yes | Sections 3 and 4 |
| 11d | Discussion — main take-away lessons | Yes | Sections 3 and 4 |
| 12 | Patient perspective | Yes | Section 2.1 (Patient perspective) |
| 13 | Informed consent — obtained | Yes | Ethics and Consent |

**Reference:** *Gagnier JJ, Kienle G, Altman DG, Moher D, Sox H, Riley D; CARE Group. The CARE guidelines: consensus-based clinical case report guideline development. J Clin Epidemiol. 2014;67(1):46–51.*
